# Uric acid-induced cardiomyocytic polyamines’ insufficience: a potential mechanism mediates cardiomyocytic injury

**DOI:** 10.3389/fendo.2025.1504614

**Published:** 2025-04-07

**Authors:** Cuiting Lin, Qiang Zheng, Haiyan Yu, Ting Wu, Lin Chen, Weihao Lin, Jianxin Pang, Yang Yang

**Affiliations:** ^1^ Department of Pharmacy, Pingshan Hospital, Southern Medical University, Shenzhen, Guangdong, China; ^2^ Department of Pharmacy, Pingshan District Peoples' Hospital of Shenzhen, Shenzhen, Guangdong, China; ^3^ Guangdong Provincial Key Laboratory of Drug Screening, School of Pharmaceutical Sciences, Southern Medical University, Guangzhou, Guangdong, China; ^4^ Neurology Department of Shenzhen Qianhai Taikang Hospital, Shenzhen, Guangdong, China; ^5^ Affiliated Foshan Maternity & Child Healthcare Hospital, Southern Medical University, Foshan, Guangdong, China

**Keywords:** polyamines, uric acid, cardiomyocytes, spermidine, spermine

## Abstract

**Introduction:**

Maintaining polyamines homeostasis is essential for cardiovascular health, whereas elevated uric acid levels are recognized as a significant risk factor for the onset and progression of cardiovascular diseases. However, the interaction between uric acid and the regulation of polyamine homeostasis has not been extensively investigated. The objective of this study was to investigate the influence of uric acid on cardiac polyamines regulation and elucidate the role of polyamines in uric acid induced cardiomyocytic injury.

**Methods:**

The *in vitro* experiments utilized H9C2 cardiomyocytes, the hyperuricemic mouse model was established via potassium oxonate and hypoxanthine. Techniques included energy metabolomics, HPLC for polyamine quantification, qPCR, ELISA, immunofluorescence, and mitochondrial membrane potential assessment using JC-1 staining, MTT cell viability analysis.

**Results:**

Uric acid treatment can alter ornithine metabolism in cardiomyocytes, revealed a potential of shifting it from the traditional ornithine cycle towards the polyamine cycle. Both ODC1 and SAT1 protein levels were up-regulated in hyperuricemic mice indicated a dysorder of polyamines homostasis. A downregulation tendency of spermidine and spermine levels were observed in cardiomyocytes under uric acid treatment. Notably, exogenous supplementation with spermidine or spermine effectively mitigated the uric acid-induced decline in cardiomyocyte viability and mitochondrial membrane potential.

**Discussion:**

Uric acid disrupts polyamine homeostasis, leading to mitochondrial dysfunction and cardiomyocyte damage. Exogenous polyamine supplementation demonstrates therapeutic potential by preserving mitochondrial integrity. These findings unveil a potential mechanism underlying uric acid-induced cardiac injury and propose polyamine replenishment as a viable intervention strategy for hyperuricemia-related cardiovascular complications.

## 1 Introduction

Hyperuricemia does not an independent disease and is frequently concomitant with other complications, including gout (1), cardiovascular diseases (2), chronic kidney disease (3), non-alcoholic fatty liver disease (4), and metabolic syndrome (5). Epidemiological evidence suggests a significant association between hyperuricemia and the onset and progression of various cardiovascular diseases, such as atrial fibrillation (6), atherosclerosis (7), cardiac hypertrophy (8), and heart failure (9), among others. The impact of uric acid on cardiovascular pathological development is closely linked to alterations in biological processes within vascular endothelial cells and cardiomyocytes (2, 10). Our prior research has demonstrated that *in vitro* stimulation with uric acid induces lipid deposition within cardiomyocytes, consequently promoting cardiomyocytic injury (11). Persistent cardiomyocytic injury is a critical factor in the initiation of ventricular remodeling (12, 13). While certain clinical evidence indicates that hyperuricemia constitutes a significant risk factor for ventricular remodeling-related conditions, there have been no documented instances of uric acid directly precipitating acute cardiac diseases in clinical practice. Consequently, we hypothesize that uric acid-induced injury to cardiomyocytes is a gradual and non-acute process. Furthermore, it is plausible that a compensatory mechanism may be operative during this process, mitigating the effects in response to uric acid.

Polyamines are endogenous aliphatic nitrogen-containing bases exhibiting significant biological activity and are ubiquitously present in living organisms. Among the polyamines, spermidine, spermine, and the precursor diamine putrescine are particularly noteworthy due to their critical involvement in cellular processes such as proliferation, growth, differentiation, and apoptosis (14, 15). Polyamines fulfill a multitude of physiological functions and are implicated in the pathogenesis and progression of various conditions, including tumors (16), cardiovascular diseases (17), neurological disorders (18), and immune system diseases (19). Our metabolic analysis revealed a significant reduction in the levels of two key metabolites in the ornithine cycle, namely ornithine and citrulline, in cardiomyocytes treated with uric acid. In contrast, the upstream metabolite arginine did not exhibit a similar trend. Therefore, we speculated that ornithine or citrulline may be redirected to an alternative metabolic pathway. Ornithine serves as a precursor for polyamines (20), undergoing conversion to putrescine, which subsequently transforms into spermidine and spermine. Notably, spermidine and spermine have been documented to play significant roles in cardiac protection. Clinical data have demonstrated a reduction in spermine levels among patients with abnormal left ventricular ejection fraction and those with congestive heart failure (17). However, previous studies have not clearly elucidated the relationship between uric acid and polyamines. It is reported that polyamines are associated with mitochondrial quantity, morphology, and activity (21, 22). In the hearts of hyperuricemic mice, abnormal mitochondrial morphology has been observed, accompanied by significantly impaired cardiac function (23).

Based on this information, we hypothesized that the endogenous polyamines decreasing is a critical mechanism of uric acid induced cardiomyocytic injury. Insufficient polyamines influence mitochondrial function thereby leads to a series of consequences. To validate these hypotheses, this study investigated the primary synthesis and metabolic enzymes of polyamines, quantified the concentrations of spermidine and spermine under uric acid stimulation, and conducted experiments involving exogenous spermine and spermidine protection. This study not only elucidates the mechanism of uric acid-induced cardiomyocyte injury from a novel perspective but also provides new pharmacological insights and potential intervention strategies for treatment.

## 2 Materials and methods

### 2.1 Cardiomyocytes culture and treatment

The H9C2 cardiomyocytes were grown at 37°C under 5% CO_2_ in Dulbeccos Modified Eagle Medium plus F12 medium (DMEM/F12) (Procell, PM150310) supplemented with fetal bovine serum10% (FBS) (Procell, 164210-50). The cardiomyocytes were dissociated and seeded at a density of 3×10^4^/cm^2^ on plates 2 days before pharmacological treatment. The uric acid (Aladdin, U105582) was premixed with the culture medium at a concentration of 15mg/dL and then added into the cells, the blank culture medium was used as control. The examinations were performed in indicated time respectively.

### 2.2 Animal model and treatment

Healthy SPF male Kunming (KM) mice, weighing 20 ± 2 g, were provided by the Laboratory Animal Center of Southern Medical University (Guangzhou, China). All animal experiments were carried out following the guidelines of the Institutional Animal Care Committee of Southern Medical University and national animal experimental ethics standards. The animals were fed standard chow for one week in a suitable experimental environment with a 12-h/12-h light/dark cycle and a controlled temperature (25 ± 1°C) and humidity (50 ± 10%). The mice were randomly divided into blank group and hyperuricemic model group. The model group received daily intraperitoneal injection of potassium oxonate (PO) 350 mg/kg and oral gavage of hypoxanthine (HX) 450 mg/kg, while the blank group received the same amount of solvent. After one week of induction, all of the mice were sacrificed, and the blood and the heart tissues were harvested for further examination.

### 2.3 Energy metabolomics analysis

The cardiomyocytes with or without uric acid treatment were treated as 2.1 indicated and then harvested after one hour treatment. The cell samples were harvested directly with a cell scraper with precooled phosphate buffered saline (PBS), followed by transfer into a 1.5 mL centrifuge tube. Then, the samples were frozen immediately and stored at -80°C. The frozen samples were sent on dry ice to MetWare Co., Ltd. (Wuhan, China) for Energy metabolomics analysis. The data was acquired using Ultra Performance Liquid Chromatography (UPLC, Waters ACQUITY H-ClassD) Tandem Mass Spectrometry (QTRAP^®^ 6500+). The UPLC analytical conditions were as follows: UPLC column, ACQUITY UPLC BEH Amide column (1.7 µm, 100 mm×2.1 mm i.d.); column temperature, 40°C; flow rate, 0.4 mL/min; injection volume, 2 µL; solvent system, water (10 mM ammonium acetate and 0.3% ammonia): 90:10 acetonitrile:water; and gradient program, 5:95 V/V at 0-1.2min, 30:70 V/V at 8min, 50:50 V/V at 9-11min, 5:95 V/V at 11.1-15min. The ESI source operation parameters were as follows: source temperature, 550°C;; ion spray voltage, 5,500 V (positive), -4,500 V (negative);curtain gas was set at 35 psi. The metabolite identification was based on MWDB (metware database), and the metabolite quantification was accomplished by using the multiple reaction monitoring (MRM) mode of triple quadrupole mass spectrometry. The mass spectrometry data was processed with the Analyst 1.6.3 and MultiQuant 3.0.3.

### 2.4 Bioinformatic data source

Using the search term “hypertrophic cardiomyopathy” and “homo sapiens” to search series in Gene Expression Omnibus (GEO) website, GSE36961 was screened out. This serious contains 39 normal control and 106 hypertrophic cardiomyopathy samples. The gene expression profiles were generated by Illumina HumanHT-12 V3.0 expression beadchip.

### 2.5 Spermidine and spermine quantification

The concentrations of spermidine and spermine in mice heart and cardiomyocytes were determined by high-performance liquid chromatography (HPLC) method. The heart tissues were collected as above mentioned and stored at -80°C immediately. The cell samples were harvested directly with a cell scraper with precooled PBS, followed by transfer into a 1.5 mL centrifuge tube. Then, the samples were frozen immediately and stored at -80°C. The frozen samples were sent on dry ice to BiotechPack (Beijing, China) for HPLC measurement.

### 2.6 Reverse transcription and real-time quantitative PCR

Total RNA was extracted by using RNAiso Plus reagent (Takara, 9109) following the protocol recommended by the manufacturer. Reverse transcription was conducted using a StarScript II First-strand cDNA Synthesis Kit-II (GenStar, A214-10) following the protocol recommended by the manufacturer. Real-time qPCR was conducted in 96-well format plates using TB Green™ Premix Ex Taq™ (Takara, RR420A) following the protocol recommended by the manufacturer. For each analysis, the mRNA level was normalized to the levels of the housekeeping gene β-actin. Information on the primers used is presented in **Supplementary Table S1**.

### 2.7 ELISA analysis

The ELISA analysis was used to quantify the concentrations of particular molecules in heart tissues. ELISA analysis were performed with commercial ELISA kits following the protocol recommended by the manufacturer. The ODC1 quantification were performed by using mouse ODC1 ELISA kit (Meimian, MM-45598M2). The SAT1 quantification were performed by using mouse SAT1 ELISA kit (Meimian, MM-46735M2).

### 2.8 Histological and immunofluorescence examination

The animals were sacrificed, and the organs were isolated and transferred to Eppendorf tubes prefilled with 4% paraformaldehyde. Paraffin-embedded tissue specimens were used to prepare 4-μm-thick sections. For immunofluorescence examination, after deparaffinization and rehydration, antigen retrieval with target retrieval solution was performed, and the sections were blocked and incubated with the respective primary antibodies overnight at 4°C. Then, the sections were washed with PBS and incubated with secondary antibodies for 1 h in the dark. Finally, the sections were stained with DAPI solution to visualize nuclei. Imaging of the heart sections was performed by Servicebio Technology Co., Ltd. (Wuhan, China). The following primary antibodies were used: ODC1 (Proteintech, 28728-1-AP), SAT1 (Proteintech, 10708-1-AP), Alexa Fluor^®^ 488-conjugated Goat Anti-Rabbit IgG (H+L) (Servicebio, GB25303).

### 2.9 Cell viability analysis

The cell viability of cardiomyocytes was assessed with MTT assays. The cardiomyocytes were treated as 2.1 indicated, while the culture medium should supplemented with 1mM aminoguanidine hydrochloride (Aladdin, A151036) to avoid exogenous spermidine and spermine degradation caused by bovine serum amine oxidase. Spermidine (Macklin, S817735) and spermine (Macklin, S817881) were premixed with the culture medium with or without uric acid and then then added into the cells. After 48 hours incubation, the culture medium was removed and replaced with 0.1 mg MTT reagent (Beyotime, ST1537) per well dissociated in 100 μL basal DMEM (Procell, PM150210). Then, the cells were incubated at 37°C for 4 hours, followed by replacement of the medium with 100 μL DMSO (Aladdin, D103272). After 10 minutes of shaking, viability was assessed by measuring the absorbance at a wavelength of 570 nm.

### 2.10 Mitochondrial membrane potential assessment

Mitochondrial membrane potential was evaluated using JC-1 staining followed by flow cytometric analysis. The cardiomyocytes were dissociated and seeded at a density of 3×10^4^/cm^2^ on plates 2 days before pharmacological treatment. The uric acid, spermidine and spermine were premixed with HBSS buffer (Procell, PB180323) before being added to the cells. The culture medium was subsequently removed from the cells, and the cells were washed twice with HBSS buffer. The prepared mixture buffers were then added to the cells. After a 1-hour incubation period, the cells were detached using Trypsin buffer and collected into 1.5 mL centrifuge tubes. Subsequently, the cells were stained with JC-1 according to the manufacturer’s protocol (Beyotime, C2003S). The stained cells were then subjected to flow cytometric analysis. The mitochondrial membrane potential was calculated by the ratio of aggregates to monomers.

## 3 Results

### 3.1 The reduction of the polyamine precursor ornithine in cardiomyocytes treated with uric acid

**Figure 1:**
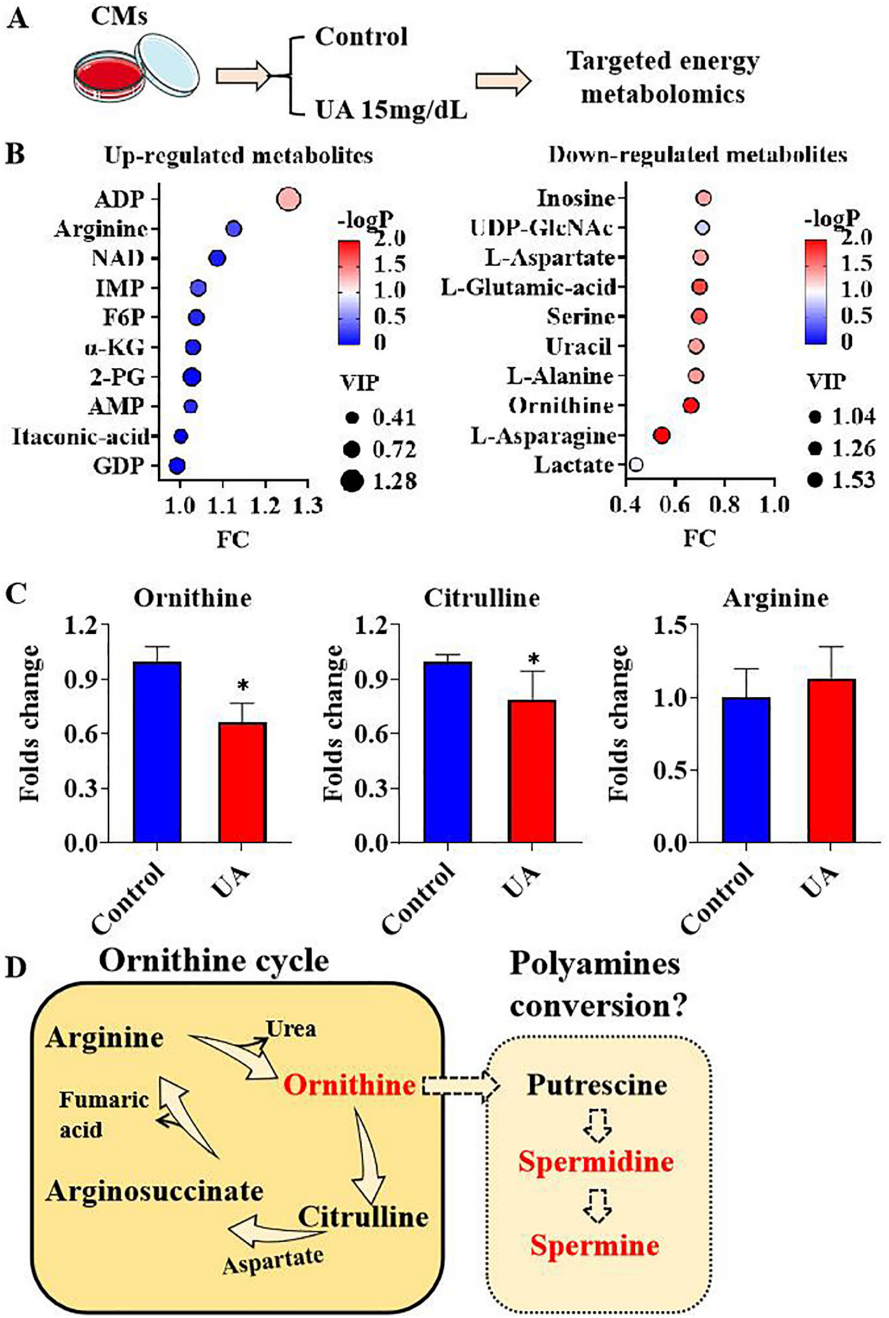
metabolites response under uric acid stimulation **(A)**. Graphic description of group information; **(B)**. Top 10 up-regulated and down-regulated metabolites under uric acid stimulation; **(C)**. Ornithine cycle components ornithine, citrulline and arginine folds change under uric acid stimulation; **(D)** The graphic hypothesis of ornithine cycle to polyamines conversion description. (n=3). * P<0.05.

The transition between physiological and pathophysiological states in cardiomyocytes is linked to alterations in cellular metabolism. To elucidate the impact of uric acid on cardiomyocyte metabolism, a targeted energy metabolomics analysis was conducted to compare metabolite concentrations between cardiomyocytes exposed to uric acid and those not exposed, as illustrated in **Figure 1A**. As demonstrated in **Supplementary Table 2**, a total of 68 metabolites were identified. The PLS-DA score scatter plot revealed distinct metabolite profiles between cardiomyocytes from the control group and those treated with uric acid (**Supplementary Figure 1A**). The values of R2Y and Q2 further indicated the model’s goodness of fit (**Supplementary Figure 1B**). Compared to the control group, the uric acid-treated cardiomyocytes exhibited significant upregulation in the top 10 metabolites, namely adenosine diphosphate (ADP), arginine, nicotinamide adenine dinucleotide (NAD), inosine monophosphate (IMP), fructose-6-phosphate (F6P), α-ketoglutaric acid (α-KG), 2-phosphoglycerate (2-PG), adenosine monophosphate (AMP), itaconic acid, and guanosine diphosphate (GDP). Conversely, the top 10 down-regulated metabolites included inosine, uridine-5’-diphosphate N-acetylglucosamine (UDP-GlcNAc), L-aspartate, L-glutamic acid, serine, uracil, L-alanine, ornithine, L-asparagine, and lactate (**Figure 1B**). We observed an imbalance in three metabolites within the ornithine cycle under conditions of uric acid stimulation. The ornithine cycle, a series of cyclic reactions occurring in the mitochondria and cytosol of cells, is responsible for synthesizing urea from ammonia and carbon dioxide. During this cycle, arginine is hydrolyzed to produce urea and ornithine, which is subsequently converted back to arginine through intermediate steps involving citrulline and argininosuccinic acid. In our metabolomics analysis, cardiomyocytes treated with uric acid exhibited significantly lower levels of ornithine and citrulline compared to the control group, while upstream arginine demonstrated an up-regulation trend (**Figure 1C**). This observation suggests a potential shift from the ornithine cycle to an alternative metabolic pathway, beginning with the reduction of ornithine. One such alternative is the polyamine biosynthesis pathway, where ornithine is sequentially converted into putrescine, spermidine, and spermine through the action of polyamine-related enzymes, thereby fulfilling various biological functions (**Figure 1D**).

### 3.2 The regulation of enzymes associated with polyamines plays a significant role in cardiac hypertrophy

**Figure 2:**
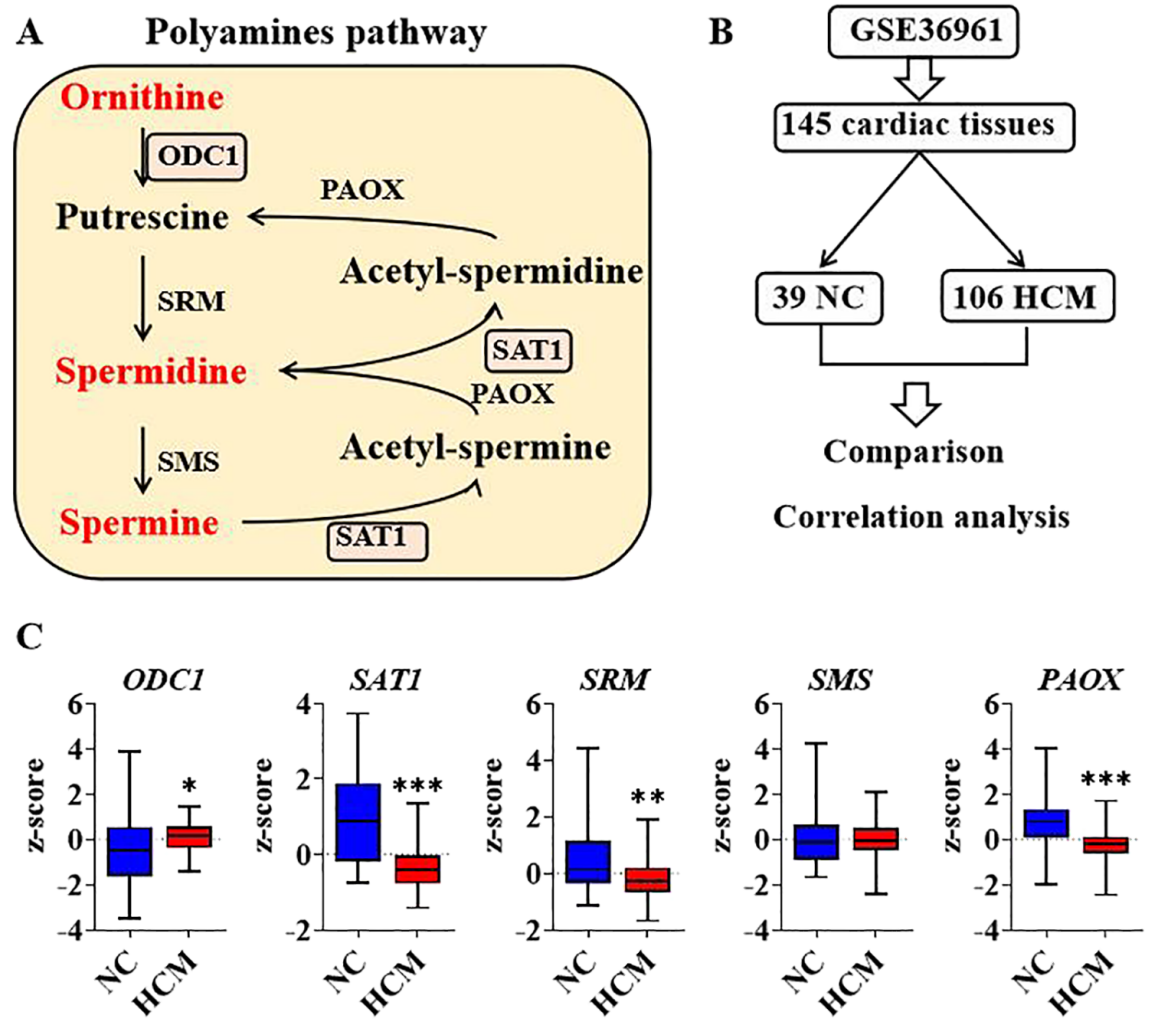
Analysis of polyamines relevant genes regulation in human cardiac hypertrophy **(A)**. Diagram of enzymes and components in polyamines pathway; **(B)**. Graphic description of GSE36961 and group information; **(C)**. Transcriptional expression of polyamines genes between negative control and hypertrophic cardiomyopathy by comparison. *P<0.05, **P<0.01, ***P<0.001.

The conversion of ornithine to spermine represents a complex and intricate biochemical pathway involving the catalytic action of multiple enzymes. Initially, ornithine undergoes a decarboxylation reaction facilitated by ornithine decarboxylase (ODC1), resulting in the production of putrescine. Subsequently, putrescine reacts with decarboxylated S-adenosylmethionine (dcSAM) under the catalysis of spermidine synthase (SRM), forming spermidine. Finally, spermidine undergoes another reaction with dcSAM, catalyzed by spermine synthase (SMS), ultimately yielding spermine. the catalytic action of spermidine/spermine N1-acetyltransferase-1 (SAT1). Subsequently, N1-acetylspermine is converted to spermidine under the influence of peroxisomal acetylpolyamine oxidase (PAOX). Moreover, spermidine can undergo a series of reactions catalyzed by both SAT1 and PAOX, ultimately resulting in the formation of putrescine (**Figure 2A**). ODC1 and SAT1 are rate-limiting enzymes in the synthesis and metabolism of polyamines. Cardiac hypertrophy represents an adaptive remodeling process that occurs in response to injury. To elucidate the relationship between polyamine homeostasis and pathological changes in the heart, a comparative analysis of transcriptional data from cardiac tissues was conducted between normal control (NC) and hypertrophic cardiomyopathy (HCM) samples. The transcriptional data were obtained from the GSE36961 dataset in the Gene Expression Omnibus (GEO) database. The dataset comprises 145 cardiac tissue samples, which can be categorized into two groups: 39 normal NC and 106 hypertrophic HCM samples (**Figure 2B**). Analysis revealed that, compared to the NC group, the transcriptional expression of ODC1 in the HCM group was significantly up-regulated, whereas the expression levels of SAT1, SRM, and PAOX were significantly down-regulated. The differences in SMS expression were not statistically significant (**Figure 2C**). These findings suggest a potential association between polyamine homeostasis and the pathological development of cardiac hypertrophy.

### 3.3 The association between polyamine-related enzymes and genes implicated in cardiac injury

**Figure 3:**
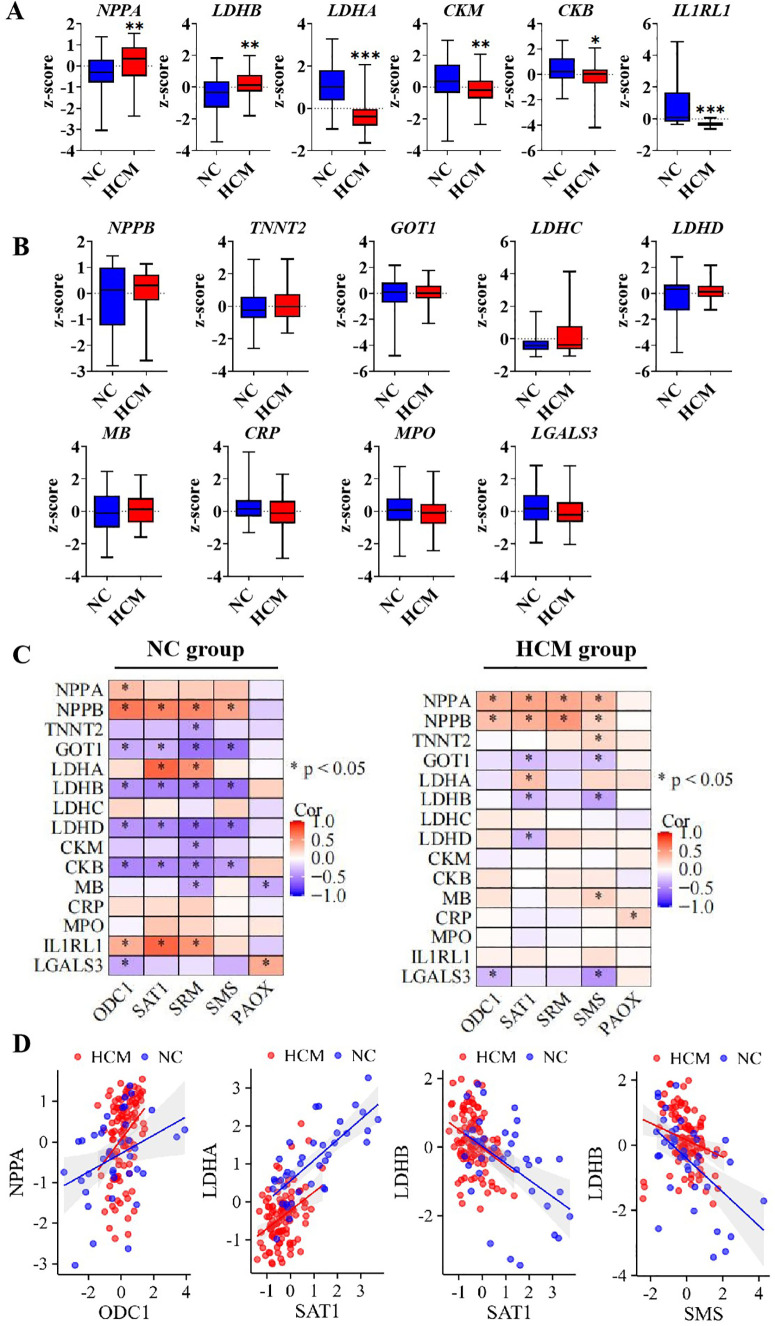
The correlation of polyamines relevant genes and cardiac injure genes expression **(A)**. The significant express genes between negative control and hypertrophic cardiomyopathy; **(B)**. The unsignificant genes between negative control and hypertrophic cardiomyopathy; **(C)**. Multiple correlation analysis between cardiac injure genes and polyamines relevant genes in NC group and HCM group; **(D)**. The liner correlation graph of significant correlation in both NC and HCM group. *P<0.05, **P<0.01, ***P<0.001.

Cardiac injury biomarkers exhibited varying sensitivity across different pathological patterns. A comparative analysis was conducted to examine the transcriptional expression of common cardiac injury biomarkers between the NC group and the HCM group. The results indicated that, compared to the NC group, the transcriptional expression of natriuretic peptide A (NPPA) and lactate dehydrogenase B (LDHB) were significantly up-regulated in the HCM group, whereas lactate dehydrogenase A (LDHA), creatine kinase M-type (CKM), creatine kinase B (CKB), and interleukin 1 receptor-like 1 (IL1RL1) were significantly down-regulated (**Figure 3A**). Other common cardiac injure biomarkers genes like natriuretic peptide B (NPPB), troponin T2 (TNNT2), glutamic-oxaloacetic transaminase 1 (GOT1), lactate dehydrogenase C (LDHC), lactate dehydrogenase D (LDHD), myoglobin (MB), C-reactive protein (CRP), myeloperoxidase (MPO) and Galectin 3 (LGALS3) showed no significant differences between NC and HCM group (**Figure 3B**). Subsequently, multiple Spearman correlation analyses were conducted to examine the relationships between polyamine-related enzymes and genes associated with cardiac injury in both the NC group and the HCM group. The results indicated a more pronounced correlation in the NC group compared to the HCM group, particularly concerning the association between polyamine-related enzymes and the LDH (lactate dehydrogenase) family (**Figure 3C**). These findings suggest that the influence of polyamines on cardiac hypertrophy may be more critical during the transition from a physiological to a pathological state, rather than during the progression of the pathological state itself. It is noteworthy that among the differentially expressed genes between NC and HCM, NPPA exhibited a significantly positive correlation with the polyamine synthesis rate-limiting enzyme ODC1. Additionally, LDHA demonstrated a significantly positive correlation with the polyamine metabolic rate-limiting enzyme SAT1, while LDHB showed a significantly negative correlation with SAT1 and SMS. These significant correlations were observed in both the NC and HCM groups (**Figure 3D**). This suggests that polyamine homeostasis may be associated with cardiac hypertrophic development.

### 3.4 The activity of polyamine synthesis and metabolic enzymes in the hearts of hyperuricemic mice

**Figure 4:**
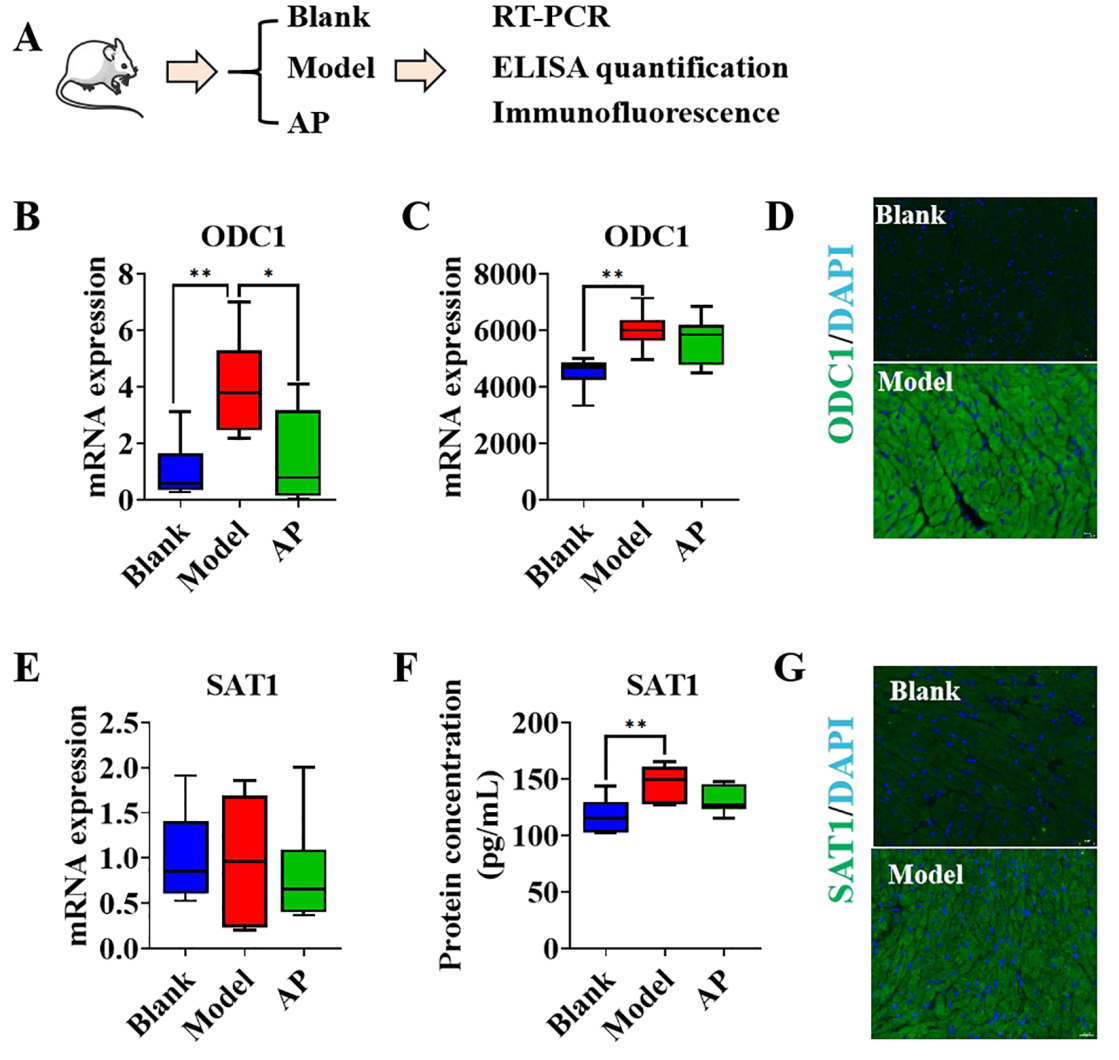
The polyamines relevant genes performance in hyperuricemic mice model **(A)**. Graphic description of group information; **(B)**. The mRNA folds change of ODC1 in the heart samples by qPCR(n=6); **(C)**. The protein concentration of ODC1 in heart samples by ELISA quantification (n=6); **(D)**. The immunofluorescence of ODC1 in mice heart, DAPI indicates nucle; **(E)**. The mRNA folds change of SAT1 in the heart samples by qPCR(n=6); **(F)**. The protein concentration of ODC1 in heart samples by ELISA quantification (n=6); **(G)** The immunofluorescence of ODC1 in mice heart, DAPI indicates nucle. *P<0.05, **P<0.01.

Our previous study indicated that long-term hyperuricemia modeling leads to hypertrophic cardiomyopathy (HCM). To investigate whether an imbalance in polyamine homeostasis exists, we established a one-week hyperuricemia model without any macroscopic pathological changes in the heart. The mice hearts were subsequently harvested for analysis, and the urate-lowering drug allopurinol was used as a positive control (**Figure 4A**). Compared to the hearts of control mice, the hearts of hyperuricemic mice exhibited significantly higher mRNA expression of ornithine decarboxylase 1 (ODC1), a trend that was mitigated by allopurinol treatment (**Figure 4B**). The protein level of ODC1 was quantified using ELISA analysis, revealing that the hearts of hyperuricemic mice exhibited a higher concentration of ODC1 compared to the control group. This trend was slightly changed with no significance following allopurinol treatment (**Figure 4C**). Immunostaining results for ODC1 corroborated the ELISA findings (**Figure 4D**). These observations suggest that cardiomyocytes may upregulate polyamine synthesis in response to hyperuricemia, potentially as a compensatory mechanism for insufficient endogenous polyamines. However, no significant differences in ODC1 mRNA expression were observed between the hearts of hyperuricemic mice and the control group (**Figure 4E**). However, the protein level of SAT1 in the hearts of hyperuricemic mice was significantly higher compared to the control group, while allopurinol treatment mitigated this increase (**Figure 4F**). The immunostaining results for SAT1 were consistent with the ELISA findings (**Figure 4G**), suggesting that polyamine metabolism is also exacerbated in response to hyperuricemia. This may be attributable to insufficient endogenous polyamines.

### 3.5 Degradation of cardiomyocyte-derived spermine and spermidine in response to uric acid

**Figure 5:**
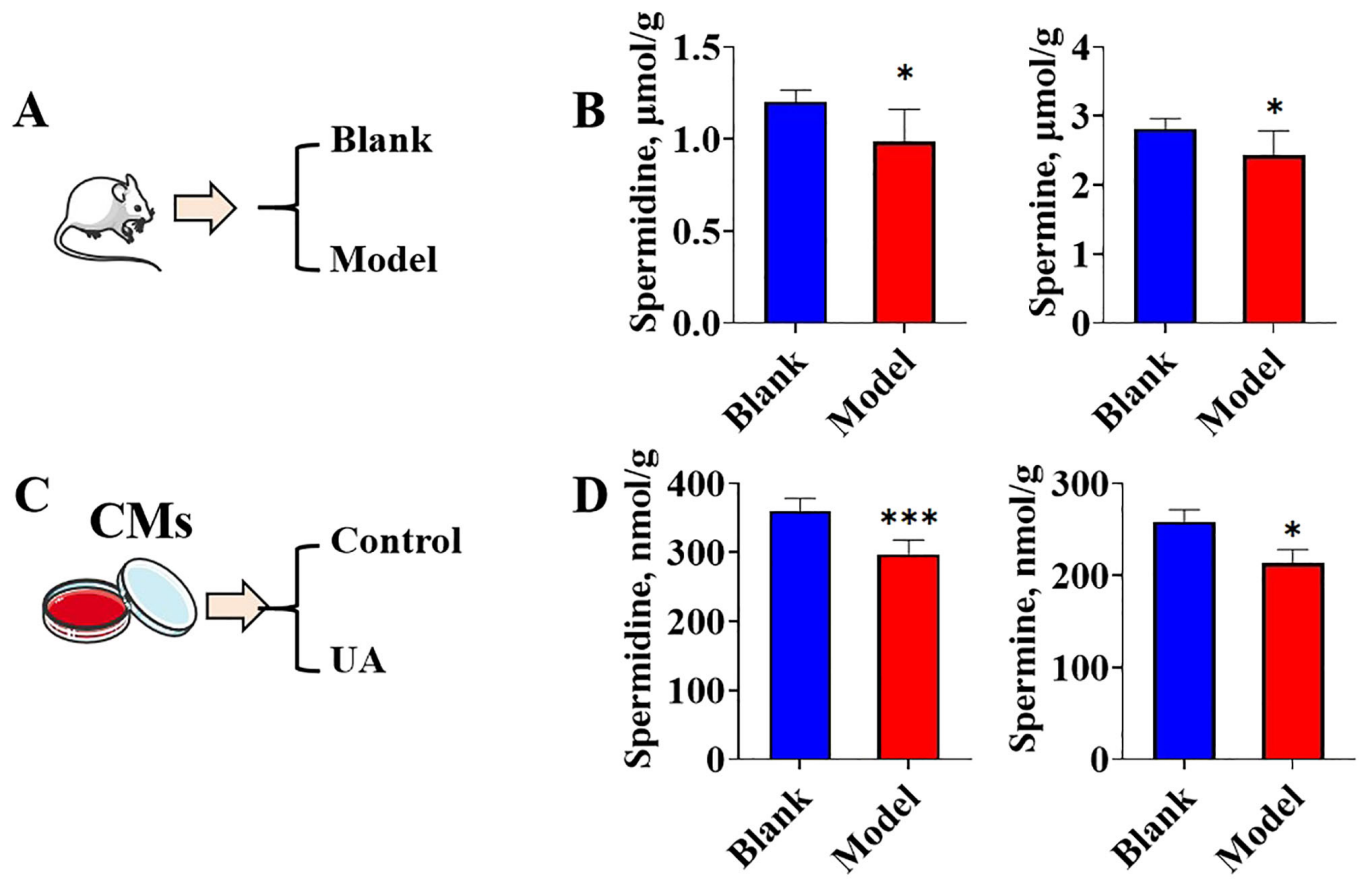
The spermidine and spermine concentrations change under uric acid stimulation **(A)**. Graphic description of animal model group information; **(B)**. spermidine and spermine concentrations in mice heart tissues (n=6); **(C)**. Graphic description of cell treatment group information; **(D)**. spermidine and spermine concentrations in cardiomyocytes (n=6). *P<0.05, ***P<0.01.

Due to the up-regulation of both the protein levels of the rate-limiting enzyme for polyamine synthesis and the metabolic rate-limiting enzyme in the hearts of hyperuricemic mice, the endogenous concentration of polyamines remains unclear and warrants further investigation. The concentrations of spermine and spermidine were quantified using HPLC. The collection of animal samples followed the procedure outlined in **Figure 5A**. Compared to the hearts of control mice, the hearts of hyperuricemic mice exhibited significantly lower concentrations of spermine and spermidine (**Figure 5B**). The cell experiments were divided into two groups: a control group without uric acid stimulation and a uric acid-treated group (**Figure 5C**). The results demonstrated that, compared to the control group, the uric acid-treated *in vitro* cardiomyocytes exhibited significantly lower concentrations of spermine and spermidine (**Figure 5D**). These findings suggest that exposure to high concentrations of uric acid, whether *in vivo* or *in vitro*, likely promotes the consumption or degradation of spermine and spermidine in cardiomyocytes.

### 3.6 Exogenous polyamines ameliorated cardiomyocyte injury induced by uric acid

**Figure 6:**
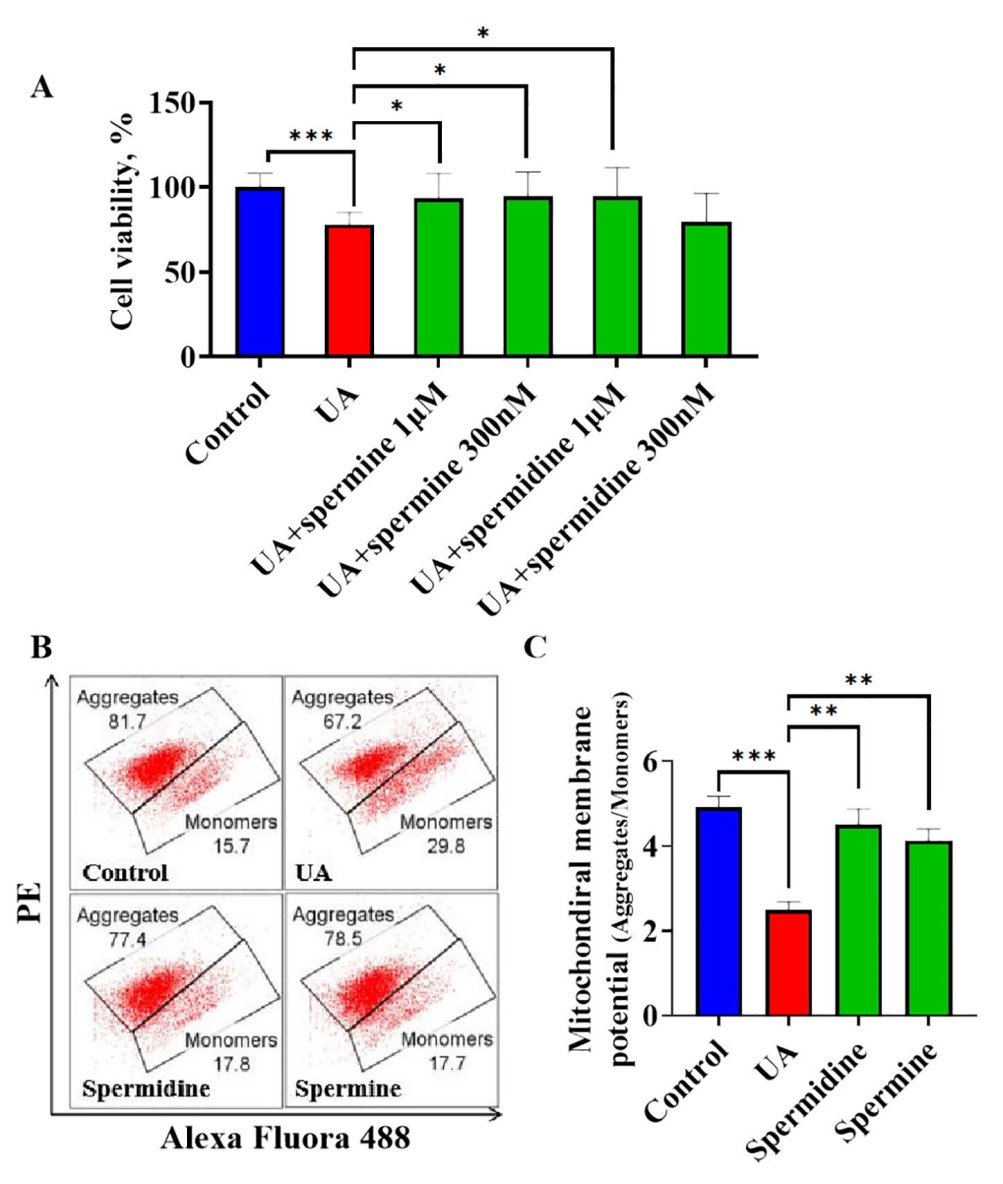
The influence of exogenous spermidine and spermine on uric acid treated cardiomyocytes **(A)**. The influence of spermine and spermidine intervention on cell viability of cardiomyocytes under uric acid stimulation (n=8); **(B)**. The representative graph of flowcytometric analysis of JC1 staining; **(C)**. The quantification of mitochondrial membrane potential by flowcytometric analysis (n=3). *P<0.05, **P<0.01, ***P<0.001.

Although uric acid promotes the reduction of endogenous polyamines, it remains unclear whether insufficient polyamine levels are responsible for inducing cardiomyocyte injury. To address this question, exogenous spermine and spermidine were administered to uric acid-treated cardiomyocytes to observe their effects. The MTT assay demonstrated that uric acid treatment significantly decreased cell viability compared to the control group, while spermine at 1μM and 300nM and spermidine at 1μM significantly attenuated uric acid induced cardiomyocytic viability decreasing, spermidine at 300nM showed no significance (**Figure 6A**). Furthermore, to assess the effects of spermine and spermidine on cardiomyocytes in the absence of uric acid treatment, these polyamines were added to the culture medium and compared to a blank control. The results indicated that neither spermine nor spermidine treatment led to a significant improvement in cell viability (**Supplementary Figure 2**). Previous study identified spermidine and spermine as metabolic regulators of mitochondrial membrane potential and highlighted uric acid as a risk factor for mitochondrial dysfunction. Consequently, we employed JC-1 detection to evaluate the influence of polyamine treatment on mitochondrial membrane potential. Therefore, JC1 detection was employed to evaluate the influence of polyamine treatment on mitochondrial membrane potential. JC1 flow cytometric analysis revealed that stimulation with uric acid resulted in an increased proportion of JC1 monomers, indicative of depolarized mitochondria, and a concomitant decrease in the proportion of JC1 aggregates, representative of normal mitochondria. This trend was reversed upon treatment with either spermine or spermidine (**Figure 6B**). Typically, the mitochondrial membrane is characterized by the ratio of aggregates to monomers. Statistical analysis of JC1 flow cytometric data revealed that uric acid significantly suppressed the mitochondrial membrane potential in cardiomyocytes. In contrast, treatment with spermine or spermidine significantly ameliorated the uric acid-induced decrease in mitochondrial membrane potential (**Figure 6C**). The results indicate that insufficient levels of endogenous polyamines are associated with uric acid-induced cardiomyocytic injury. Supplementation with exogenous spermine and spermidine has been shown to protect cardiomyocytes from uric acid-induced injury, a mechanism that is linked to the maintenance of mitochondrial membrane potential.

## 4 Discussion

Serum uric acid levels have been associated with various cardiovascular diseases. Clinical studies have indicated a significant correlation between uric acid levels and ventricular wall thickness across different populations (24–26). Our previous animal experiments demonstrated that, following eight weeks of hyperuricemia induction, KM mice exhibited increased left ventricular wall thickness (27). Ventricular remodeling is generally considered an adaptive response to cardiac injury, aimed at preserving cardiac function. In recent years, research has demonstrated that uric acid induces cardiomyocyte injury through multiple mechanisms, including the generation of reactive oxygen species (10), insulin resistance (28), regulation by non-coding RNAs (29), endoplasmic reticulum stress (30), and inflammasome activation (31), among others. Upon experiencing injury, cardiomyocytes trigger a series of compensatory mechanisms such as the augmentation of myocardial cell tensile force. This augmentation seeks to enhance the contractile capacity of the heart, thereby ensuring or increasing cardiac output and maintaining normal systemic circulation. Structurally, this compensatory process is often associated with significant cardiac remodeling, particularly characterized by hypertrophic alterations in myocardial tissue (32). Therefore, the critical mechanism driving the exacerbation of myocardial hypertrophy in hyperuricemia is rooted in the compensatory response of cardiomyocytes following uric acid-induced damage. Our *in vitro* metabolomics analysis indicates that exposure to a high-uric acid environment significantly diminishes intracellular levels of ornithine, a precursor of polyamines. Further investigation revealed that the concentrations of spermidine and spermine in the cardiac tissue of hyperuricemic mice were markedly lower compared to those in the normal control group. Similarly, the concentrations of spermidine and spermine in cardiomyocytes subjected to elevated uric acid were significantly reduced compared to the unexposed control group. Collectively, these findings indicate that the observed decrease in endogenous spermidine and spermine levels constitutes a reactive response in cardiomyocytes to uric acid stimulation.

Currently, research investigating the correlation between uric acid and polyamine homeostasis remains largely unexplored. Spermidine and spermine, which are considered essential polyamine compounds, are ubiquitously present in various tissues and organs of biological entities. These polyamines play crucial roles in protecting and regulating the functions of these tissues and organs (33, 34). Under typical physiological conditions, the natural decline of spermidine and spermine is commonly considered a hallmark of age-related processes (35, 36). Notably, research has suggested that timely supplementation with spermidine and spermine may offer potential benefits for delaying aging (37, 38). Previous studies have shown that uric acid levels in mouse heart tissue increase with advancing age (39), a trend that contrasts with the age-related decline in polyamine levels, suggesting a possible intrinsic connection between these two compounds. In our prior research, we observed that cardiac cells experienced a certain damage when exposed to uric acid; however, the extent of this damage was not severe (11). This observation suggests the existence of a compensatory mechanism within the organism that mitigates and regulates the response to uric acid stimulation, similar to mechanisms implicated in the aging process. Additionally, experimental data demonstrated a reduction in endogenous levels of spermidine and spermine in cardiomyocytes subjected to uric acid. Based on these findings, we speculate that cardiomyocytes may utilize the consumption of spermidine and spermine as a potential self-protective strategy. Subsequent experiments demonstrated that exogenous supplementation with spermidine or spermine to counteract polyamine depletion significantly ameliorated uric acid-induced myocardial cell damage, thereby providing robust experimental evidence to support our hypothesis. These findings not only enhance our understanding of the interaction between uric acid and polyamine homeostasis but also suggest new avenues for exploring preventive and therapeutic strategies for aging and related diseases in the future.

In the field of cardiovascular research, spermine and spermidine have demonstrated cardioprotective effects against various forms of injury, including diabetic cardiomyopathy (40), ischemia/reperfusion injury (41), and age-related cardiac decline (42). The bioinformatics database revealed that heart tissues from patients with HCM exhibited significantly higher gene expression of ODC1 and lower expression of SAT1 compared to NC, indicating a trend towards enhanced polyamine synthesis and reduced degradation. In our hyperuricemic mice model, both the protein and gene levels of ODC1 were up-regulated, suggesting increased synthesis to compensate insufficient polyamines. Although the protein level of SAT1 was also up-regulated, the gene expression did not reach statistical significance, possibly due to the limited sample size. We speculated that the up-regulation of SAT1 protein may contribute to the degradation of spermine and spermidine. Supplementation with spermidine has been shown to enhance cardiac mitochondrial number, morphology, biogenesis, and function (21, 43). Spermine modulates calcium transport in cardiomyocytes, thereby affecting mitochondrial function (44, 45). Studies have reported that cardiac mitochondrial morphology is compromised in uricase knockout hyperuricemia mouse models (23). Mitochondria, as essential organelles for the oxidation of glucose and fatty acids within cells, are critical for the energy supply to cardiomyocytes. Disruptions to their structure and function can significantly impact the energetic status of these cells. Research has shown that uric acid can induce insulin resistance and disrupt fatty acid metabolism in cardiomyocytes (11, 28). Metabolic conditions that are closely linked to mitochondrial metabolic dysfunction. JC1, a widely used fluorescent dye, is an effective tool for evaluating changes in mitochondrial membrane potential. Compromised mitochondrial structure and function often result in alterations in membrane potential, which are indicated by a shift in JC1 fluorescence from red to green. Flow cytometry analysis of JC1-stained samples offers an indirect yet quantitative assessment of alterations in mitochondrial membrane potential. Our findings indicate that treatment with spermine and spermidine substantially mitigates the reduction in mitochondrial membrane potential observed in cardiomyocytes subjected to uric acid induction. This finding not only enhances the comprehension of the mechanisms by which spermine and spermidine ameliorate uric acid-induced cardiac injury through mitochondrial protection but also posits that deficiencies in endogenous polyamine levels may contribute to the reduced adaptive capacity of cardiomyocytes in response to uric acid stimulation.

In summary, our study reveals a critical finding: uric acid induces polyamine depletion in cardiomyocytes, underscoring that endogenous polyamine deficiency is inadequate to mitigate the myocardial damage caused by uric acid. This pathological mechanism is likely intricately linked to mitochondrial dysfunction. This study not only provides new insights into the mechanisms underlying uric acid-mediated cardiac injury but also introduces a novel perspective and theoretical foundation for polyamine supplementation as a potential preventive strategy to mitigate cardiac damage associated with hyperuricemia.

## Data Availability

The data that supporting the finding of this article will be made available by the authors, without undue reservation. The datasets presented in this study can be found in Gene Expression Omnibus, GSE36961 and **Supplementary Materials**.
