# Supplementary material for: Uric acid-induced cardiomyocytic polyamines’ insufficience: a potential mechanism mediates cardiomyocytic injury
Source: Front Endocrinol (Lausanne). 2025 Apr 7;16:1504614. doi: 10.3389/fendo.2025.1504614 (PMC12009720; doi:10.3389/fendo.2025.1504614)
Supplement: Supplementary file 1 [file DataSheet1.pdf]

Supplementary Material

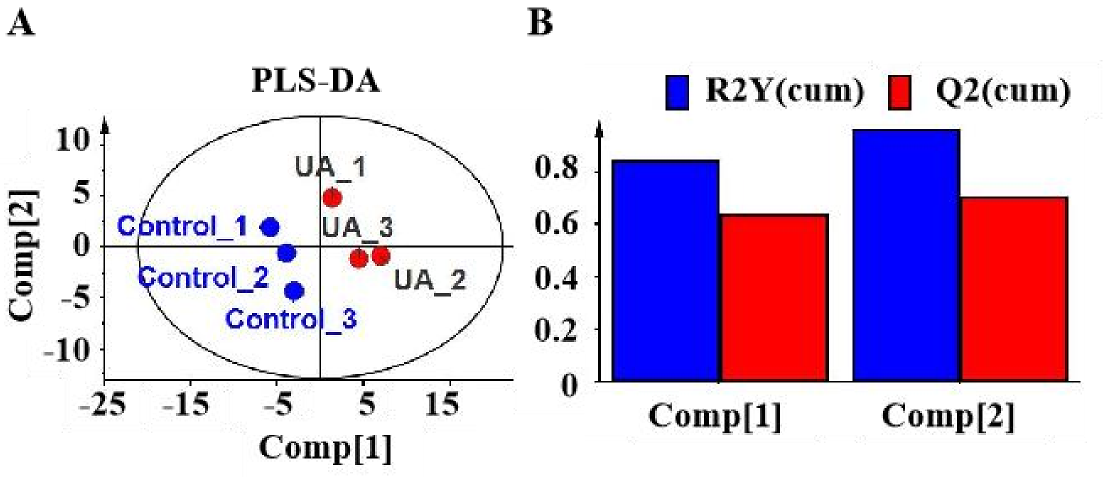

Supplementary Figure 1. basic information of metabolimics, related to Figure 1.

A. PLS-DA score scatter plot showing the separation of control group and uric acid group; B. The summary of fit plot reflects the goodness of the model.

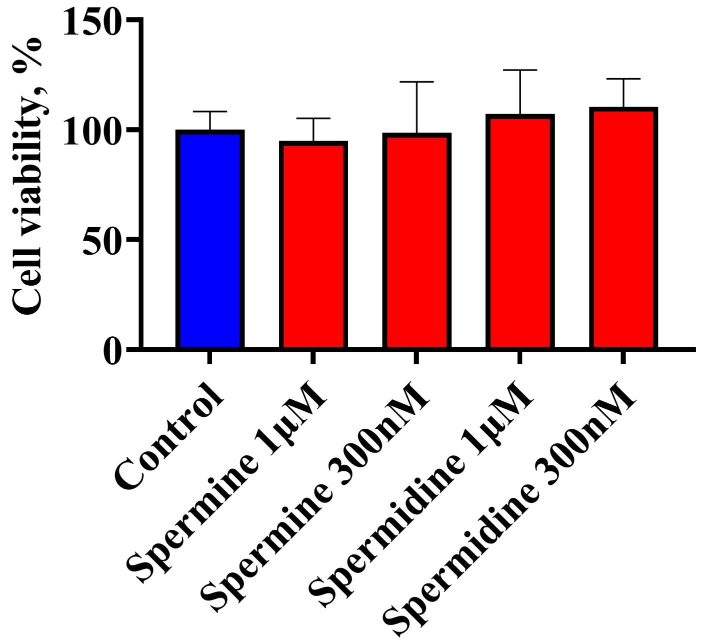

Supplementary Figure 2. The influence of exogenous spermidine and spermine on cardiomyocytic cell viability without uric acid treatment (n=8), related to Figure 6.

Supplementary Table 1 List of primers used for real-time qPCR

Gene	Sequence (5'-3')	
ODC1	Forward	GACGAGTTTGACTGCCACATC
	Reverse	CGCAACATAGAACGCATCCTT
SAT1	Forward	GAGAACACCCCTTCTACCACT
	Reverse	GCCTCTGTAATCACTCATCACGA
β-actin	Forward	GTGACGTTGACATCCGTAAAGA
	Reverse	GCCGGACTCATCGTACTCC

Supplementary Table 2 List of targeted energy metabolomics data

Compounds	**CON1**	**CON2**	**CON3**	**UA4**	**UA5**	**UA6**
2-Phospho-D-glyceric acid	850.18	735.24	275.25	912.50	533.01	419.40
3-phenyllactic-acid	0.69	0.63	0.49	0.58	0.46	0.51
3-phosphoglycerate	551.52	571.73	466.70	630.34	388.57	471.13
6-Phosphogluconic-acid	476.41	461.07	444.13	407.46	396.02	511.25
Acetyl-CoA	31.02	31.05	34.39	26.84	31.27	28.25
Adenine	2206.28	2152.98	1957.05	1952.07	1640.14	1756.39
ADP	253.74	268.96	284.79	310.35	318.83	276.07
Alpha-Ketoglutaric-Acid	35.28	35.78	42.13	33.65	38.54	30.04
AMP	680.56	730.66	656.40	688.39	663.62	331.35
Arginine	22.33	23.41	31.61	31.50	33.10	26.29
Argininosuccinic-acid	5.99	6.21	7.11	6.05	6.33	7.37
ATP	152.36	110.77	132.61	127.42	105.77	43.01
c-di-AMP	119.65	121.93	136.70	101.17	122.86	107.67
cis-Aconitic-acid	8.00	8.00	9.45	7.03	7.98	8.25
Cyclic-AMP	2.81	2.76	3.02	2.44	2.69	2.72
dAMP	60.98	71.73	64.55	53.18	56.39	37.88
dCMP	25.64	35.43	27.56	25.04	25.33	12.42
D-Erythrose 4-phosphate	145.21	135.75	150.88	131.02	114.97	153.71
D-Fructose-6-phosphate	101.09	77.19	76.29	87.36	86.52	161.31
D-Glucose-1-phosphate	104.14	80.88	85.90	87.62	85.88	154.80
D-Glucose-6-phosphate	122.10	105.17	99.54	101.69	105.41	215.97
Dihydroxyacetone-phosphate	353.17	328.79	392.66	344.07	336.15	365.49
D-Ribulose-5-phosphate	523.43	566.30	618.34	553.50	422.25	819.34
dTMP	106.76	125.24	94.99	92.27	81.76	71.18
dUMP	40.68	39.71	35.74	31.78	27.91	19.69
Flavin-mononucleotide	38.55	37.19	38.19	36.63	35.15	32.01
Fructose-1,6-bisphosphate	158.51	117.74	142.14	139.09	115.16	181.52
Fumaric-acid	53.48	51.56	50.18	48.55	46.06	40.65
Glutamine	370.86	363.06	401.77	322.93	250.32	743.92
Glyceraldehyde-3-phosphate	411.11	363.85	440.47	382.54	355.60	409.42
Glycerol-3-phosphate	43.98	48.76	44.72	40.70	36.55	18.80

Guanosine	124.94	94.69	124.69	124.82	88.42	54.37
Guanosine-diphosphate	55.07	56.32	67.83	49.21	59.18	52.76
IMP	517.54	594.22	528.64	564.93	539.98	282.57
Inosine	9.24	8.57	11.20	7.87	6.81	11.86
Itaconic-acid	6.64	6.42	7.62	5.88	6.67	7.03
Lactate	97.02	42.78	48.43	27.90	22.10	24.75
L-Alanine	372.42	341.11	323.96	290.86	172.98	285.14
L-Asparagine	57.00	52.02	49.46	32.84	28.35	147.64
L-Aspartate	956.30	895.05	776.94	764.12	526.29	679.01
L-citrulline	679.45	700.58	652.64	656.92	457.43	610.72
L-Cystine	5.59	5.63	4.66	4.94	4.06	7.25
L-Glutamic-acid	2633.52	2555.72	2230.48	2020.56	1494.51	1710.83
L-Leucine	1339.62	1269.35	1342.59	1191.89	863.92	1314.90
Lysine	451.26	402.35	417.57	366.20	294.42	314.77
Malic-acid	115.58	109.62	97.83	94.39	85.88	75.48
NicotinaMide-adenine-dinucleotide(NAD)	174.38	175.02	271.08	321.39	154.62	194.99
Ornithine	200.22	174.72	175.52	143.41	114.55	114.71
Phosphoenolpyruvic-acid	501.68	505.53	363.25	385.73	377.89	203.10
Phosphorylethanolamine	334.21	276.27	314.19	268.98	223.96	294.84
Pyruvic-acid	26.76	22.80	17.71	23.85	15.69	9.04
Sedoheptulose-7-phosphate	63.97	68.15	74.11	56.54	67.20	60.94
Serine	936.47	850.92	816.30	728.06	558.66	745.79
Succinic Acid	40.22	41.63	46.85	35.15	39.29	41.10
Threonine	351.15	322.77	299.84	294.99	209.20	276.58
Trehalose-6-phosphate	5.60	5.68	6.56	5.00	4.80	5.60
Tyrosine	393.71	372.62	357.85	348.21	247.91	344.94
UDP-GlcNAc	1376.58	1566.45	932.52	1126.30	819.15	817.60
UMP	566.75	552.76	480.69	554.24	414.93	251.36
Uracil	2434.39	2026.97	2145.34	1911.37	1294.40	1463.86
Xyluose-5-phosphate	591.47	621.92	683.83	585.23	457.33	891.69
